# Disruption of the thyroid hormone system and patterns of altered thyroid hormones after gestational chemical exposures in rodents – a systematic review

**DOI:** 10.3389/fendo.2023.1323284

**Published:** 2024-01-30

**Authors:** Isabel Forner-Piquer, Asma H. Baig, Andreas Kortenkamp

**Affiliations:** Centre for Pollution Research and Policy, Department of Life Sciences, College of Health, Medicine and Life Sciences, Brunel University London, Uxbridge, United Kingdom

**Keywords:** thyroxine, T4, thyroid stimulating hormone, TSH, endocrine disruptors, systematic review

## Abstract

We present a comprehensive overview of changes in thyroxine (T4) and thyroid stimulating hormone (TSH) serum concentrations after pre-gestational, gestational and/or lactation exposures of rodents to various chemicals that affect the thyroid hormone system. We show that T4 and TSH changes consistent with the idealized view of the hypothalamic-pituitary-thyroid (HPT) feedback loop (T4 decrements accompanied by TSH increases) are observed with only a relatively small set of chemicals. Most substances affect concentrations of various thyroid hormones without increasing TSH. Studies of altered T4 concentrations after gestational exposures are limited to a relatively small set of chemicals in which pesticides, pharmaceuticals and industrial chemicals are under-represented. Our risk-of-bias analysis exposed deficits in T4/TSH analytics as a problem area. By relating patterns of T4 – TSH changes to mode-of-action (MOA) information, we found that chemicals capable of disrupting the HPT feedback frequently affected thyroid hormone synthesis, while substances that produced T4 serum decrements without accompanying TSH increases lacked this ability, but often induced liver enzyme systems responsible for the elimination of TH by glucuronidation. Importantly, a multitude of MOA leads to decrements of serum T4. The current EU approaches for identifying thyroid hormone system-disrupting chemicals, with their reliance on altered TH serum levels as indicators of a hormonal mode of action and thyroid histopathological changes as indicators of adversity, will miss chemicals that produce T4/T3 serum decreases without accompanying TSH increases. This is of concern as it may lead to a disregard for chemicals that produce developmental neurotoxicity by disrupting adequate T4/T3 supply to the brain, but without increasing TSH.

## 1 Introduction

The thyroid hormone system is involved in the regulation of many vital processes, including lipid metabolism and brain development. These processes are critically dependent on the availability of thyroid hormones (TH) in target tissues, at the correct amount and at the correct time. Mis-timed delivery and over- or under-supply of TH can have adverse and irreversible consequences (1). The complexity of the thyroid hormone system is considerable, involving multiple steps including receptor binding, transport, cellular uptake and hormone conversion-steps controlling the formation of the active hormone, tri-iodothyronine (T3) in target cells. This complexity poses enormous challenges for the identification of chemicals capable of disrupting the proper functioning of the system, here termed thyroid hormone system-disrupting chemicals (THSDC).

Current approaches for the identification of THSDC in the European Union (EU) (2) rely on determinations of altered TH serum levels (T4 and T3), which are taken as indicators of a hormonal mode of action. In addition, adverse effects need to be demonstrated. In the absence of other signs of adversity (e.g. neurodevelopmental toxicity), histopathological changes in the thyroid gland are seen as satisfying the adversity criterion. Such changes are mainly the result of elevated TSH levels.

The rationale of this approach can be traced to concepts of target organ toxicity for the thyroid, and to an idealized view of the canonical hypothalamic-pituitary-thyroid (HPT) axis feedback loop. In this view, insufficient TH output by the thyroid stimulates the release of thyrotropin-releasing hormone (TRH) in hypothalamic neurons and of TSH in pituitary thyrotropes. In turn, TSH induces the thyroid to produce TH, ultimately restoring T4 and TSH to normal serum concentrations. Persistently elevated TSH concentrations can lead to histopathological changes in the thyroid, shifting the gland to a trajectory from follicular cell proliferation to certain cancers (3). Other factors, including iodine deficiency or mutations can also contribute to these changes.

T4 and TSH serum levels consistent with this idealized view of the HPT axis are, however, not always observed. The most striking examples include chemicals such as PCBs, PBDEs and perfluorinated compounds which cause large T4 serum decrements without the expected increases in TSH levels (4–8). Although the reasons for these enigmatic patterns remain to be fully elucidated, they suggest that the underlying mechanisms of action involve parts of the thyroid hormone system that are extraneous to the HPT axis. In any case, the concern is that current EU approaches for the testing and evaluation of THSDCs, with their reliance on adverse changes in thyroid gland histopathology, will miss chemicals which exhibit patterns of hormonal changes incompatible with the idealized view of the HPT axis, yet can disrupt the thyroid hormone system, with negative consequences for healthy brain development.

The aim of our study is to produce a comprehensive overview of the T4 and TSH changes in rodents seen after gestational/lactational exposures to a wide variety of chemicals. Relying on the peer-reviewed scientific literature only, we wanted to compare the effects of different chemicals to discover the most frequently observed patterns, to establish whether different patterns were observed with the same chemical, to compare the effects between dams and pups and to understand how the ways in which the thyroid hormone system was perturbed (here referred to as mode of action, MoA) relate to these patterns. To achieve this, we conducted a systematic evidence mapping with confidence rating of experimental studies. One outcome of this work will be to provide an improved basis for regulatory bodies to consider a wider range of MoA in the assessment of chemicals capable of disrupting the thyroid hormone system.

## 2 Materials and methods

### 2.1 Literature search and screening

The systematic review methodology was developed following the COSTER recommendations and reported in accordance with the Preferred Reporting Item for Systematic Reviews and Meta-Analysis (PRISMA) checklist (9). The detailed protocol for the present systematic review can be found in the open-access repository Zenodo, uploaded in 2021 (https://zenodo.org/record/5528557#.Y_dAOybP02w). The initial protocol uploaded to the repository was written to compile data for humans and laboratory animals (adults and offspring). However, after careful review of all the studies obtained, we decided to first focus on pre-gestational, gestational and lactational studies in rodent models. Due to the large numbers of articles, we left epidemiological data and rodent adult studies for subsequent systematic reviews.

The literature search for peer-reviewed articles was conducted in 3 scientific databases: PubMed, Web of Science and Scopus, using a search strategy with terms describing the thyroid hormone system and endocrine-disrupting chemicals to capture all pertinent information on the effects of chemicals on T4/TSH. Seven references were added through recommendation by experts, and these were designated “*ad hoc*” (AD) studies.

An initial pilot search was conducted on September 2020 with an update in July 2021. The PECO statement (Population, Exposure, Comparator, Outcome) was developed to frame the research question and the subsequent screening of the studies (**Table 1**).

**Table 1:** PECO statement.

		Inclusion criteria	Exclusion criteria
Populations	Laboratory mammalian species	Mammalian species	Non-mammalian test species such as fish or amphibians. Marine mammals
Exposures	All chemical classes: chemicals with hormonal activity – natural and man-made	Administered by gavage, drinking water or diet, with at least 2 dose groups	Administered subcutaneously or intraperitoneally; only 1 dose group
Comparators	Groups with no chemical exposure or exposure to vehicle	Control group (same species as exposure group(s)	No control group
Outcomes	Thyroid hormone measurements	Total and free T4 Total and free T3 TSH	No T4 measurements No TSH measurements

After an initial pilot study, the search string was refined with names of the target chemicals, however, this was modified as we found that such a search strategy could not capture all the literature which may be of relevance. Studies collected in this way were included in the systematic review when they complied with the PECO statement and our inclusion criteria. In the end, we built a final search string which relied on items commonly known to contain potential endocrine-disrupting chemicals (EDCs), avoiding specific names of chemicals. The list of items included was based on the information supplied by the National Institute of Environmental Health Sciences and Endocrine Society websites (10, 11) and (12, 13). We did not apply time limits in our search. The language of the publications was restricted to English. Detailed search terms are presented in **Supplementary Material 1**.

The systematic review process was managed using the freely available online tool CADIMA (https://www.cadima.info/index.php). Two team members worked independently on the merged list of records from Scopus, Web of Science and Pubmed to conduct title and abstract screening, followed by full-text screening. A consistency check of a subsample of 200 randomly selected studies was performed, in which we achieved a kappa value of 0.55, considered as “fair” based on Cadima criteria. Any potential conflicts among both screeners during the title/abstract or full-text screening were resolved after discussion or by inclusion of a third reviewer.

Articles with no full-text access, book chapters, clinical trials, reviews, systematic reviews, meta-analysis, conference proceedings, opinion articles or letters to editor were excluded. When different studies reported the same research data, we selected the study reporting the most complete dataset.

Only peer-reviewed studies were selected for data extraction and study evaluation. We included all the experimental studies (*in vivo*) with rodent models (rat and mouse) that measured the levels of T4 (total and/or free) and TSH in serum/plasma of pups/fetus and dams, and pups/fetus or dams alone after chemical administration. Studies with non-mammalian species or marine mammals were excluded, as were studies with chemical administration outside the pre-gestational, gestational or lactational periods. We also excluded studies that used mixtures of different chemicals or when the chemicals were administered by injection, as this route evades liver metabolism. We focused on studies in which individual compounds were tested at 2 or more doses, as studies with only one tested dose may suffer from a higher chance of overlooking TH/TSH dose-response effects. Although studies that used the test chemicals at only one dose did not meet our eligibility criteria, we nevertheless considered their outcomes to investigate whether they revealed response patterns consistent with eligible studies.

### 2.2 Data extraction and synthesis

Data from the studies were extracted into a template adapted from Martin et al. (14). The corresponding data extraction template can be found in **Supplementary Material 2**. Briefly, we extracted the following data:

• Meta data (Article title, authors, publication year, journal name, funding source, funding source category).

• Information about the study:

- Animal model (species, strain, Latin name, age at the beginning/end of the treatment, age at the time the TH/TSH were measured).
- Study design (duration, exposure concentration, exposure regimen, dosing route, inclusion of negative and positive control, thyroid histopathology, hepatic T4-UDPGT, measurement method for T4, measurement method for TSH, biological sample measured, statistical methods).
- Chemical characterization (chemical name, acronym, CAS, chemical class, chemical uses, source of the chemical, purity, vehicle, chemical detection: tissue and method).
- Hormone measurements in pups and dams: T4, FT4, TSH (increase “∧”, decrease “∨”, no change “–” and percentage of the change in the treatment groups with respect to the control group).

Percentage changes of T4, fT4 and TSH were calculated with the following formula:


[(level of TH in experimental group–level of TH in control group/level of TH in the control group) ×100].

When the TH/TSH data were in the form of plots or images, the freely available online tool WebPlotDigitizer (15) was used to extract the data. Where an article reported TH/TSH levels for more than one chemical, we recorded one entry for every chemical in the data extraction template.

As one of our goals was to elucidate relations between potential MoA and TH/TSH patterns, we retrieved data on other endpoints related to the TH system, including:

- Activity of T4-UDPGT, an enzyme in charge of hepatic catabolism of thyroid hormones.
- Thyroid histopathology.
- Gene and/or protein markers related to the TH axis, for example:
  - Liver metabolism: UDP-glucuronosyltransferases (UGTs), sulfotransferases (SULTs), cytochromes P450s (CYPs), constitutive androstane receptor (CAR), pregnane X receptor (PXR),
  - Synthesis: sodium-iodide cotransporter (NIS or SLC5a5), thyroid peroxidase (TPO), dual oxidases (DUOXs), thyroglobulin (TG), deiodinases (DIOs).
  - Regulation: Thyroid receptors (TRs), TSH receptor (TSHr), thyrotropin-releasing hormone (TRH), thyroid hormone responsive genes (Thrsp or Spot14, ME1, Mdra1), thyroid transcription factors (Nkx2.1, TTF-1, PAX8).
  - Distribution, transport and binding: Monocarboxylate transporter 8 (MCT8), organic anion transporting polypeptides (OATPs), pendrin, transthyretin (TTR).
  - Enzymatic activity: TPO, DIOs, 7-Ethoxyresorufin-O-deethylase (EROD), 7-pentoxyresorufin O-dealkylase (PROD), 7-benzyloxyresorufin oxidation (BROD), 7-methoxyresorufin O-demethylation (MROD).
  - Binding activity/affinity of different TH system components: TTR, TRs.
  - Levels of T4 in foetal brain and liver.
  - Neurological outcomes in offspring (e.g. motor activity, heterotopia, neurotransmitter alterations).

In addition, we explored the CompTox Chemicals Dashboard database (https://comptox.epa.gov/dashboard/) to retrieve information on EDC bioactivity from the *in vitro* TH assays (shown in **Table 2**) and the EFSA report on the establishment of cumulative assessment groups of pesticides (16) to collect information about the effect of pesticides on the thyroid.

**Table 2:** TH-related assays from CompTox.

TH element	Name of the assay	Species	Tissue (Cell line)	Assay function type	Signal direction	Intended target type	Biological process target	Source
TRHr	TOX21_TRHR_HEK293_Agonist	Human	Kidney (HEK293)	Agonist	Gain	Protein (Receptor)	Receptor activation	TOX21
	TOX21_TRHR_HEK293_Antagonist	Human	Kidney (HEK293)	Antagonist	Loss	Protein (Receptor)	Receptor activation	TOX21
TSHr	TOX21_TSHR_HTRF_Agonist_ratio	Human	Kidney (HEK293T)	Ratio	Gain	Protein (Receptor)	Regulation of transcription factor activity	TOX21
	TOX21_TSHR_HTRF_Antagonist_ratio	Human	Kidney (HEK293T)	Ratio	Loss	Protein (Receptor)	Regulation of transcription factor activity	TOX21
TR	TOX21_TR_LUC_GH3_Agonist	Rat	Pituitary (GH3)	Reporter gene	Gain	Protein (Receptor)	Regulation of transcription factor activity	TOX21
	TOX21_TR_LUC_GH3_Antagonist	Rat	Pituitary (GH3)	Antagonist	Loss	Protein (Receptor)	Regulation of transcription factor activity	TOX21
TPO	CCTE_Simmons_AUR_TPO_dn	Rat	Thyroid	Binding	Loss	Protein (Enzyme)	Regulation of catalytic activity	CCTE
NIS	CPHEA_Stoker_NIS_Inhibition_RAIU	Human	Kidney (HEK293T)	Transporter	Loss	Protein (Transporter)	Regulation of transporter activity	CPHEA_STOKER
IYD	CCTE_GLTED_hIYD_dn	Human	Cell-free	Enzymatic activity	Loss	Protein (Enzyme)	Regulation of catalytic activity	CCTE_GLTED
DIO1	CCTE_GLTED_hDIO1_dn	Human	Cell-free	Enzymatic activity	Loss	Protein (Enzyme)	Regulation of catalytic activity	CCTE_GLTED
DIO2	CCTE_GLTED_hDIO2_dn	Human	Cell-free	Enzymatic activity	Loss	Protein (Enzyme)	Regulation of catalytic activity	CCTE_GLTED
DIO3	CCTE_GLTED_hDIO3_dn	Human	Cell-free	Enzymatic activity	Loss	Protein (Enzyme)	Regulation of catalytic activity	CCTE_GLTED
SULT2a1	LTEA_HepaRG_SULT2A1_dn	Human	Liver (HepaRG)	Reporter gene	Loss	RNA (mRNA)	Regulation of transcription factor activity	LTEA
	LTEA_HepaRG_SULT2A1_up	Human	Liver (HepaRG)	Reporter gene	Gain	RNA (mRNA)	Regulation of transcription factor activity	LTEA
UGT1a1	LTEA_HepaRG_UGT1A1_dn	Human	Liver (HepaRG)	Reporter gene	Loss	RNA (mRNA)	Regulation of transcription factor activity	LTEA
	LTEA_HepaRG_UGT1A1_up	Human	Liver (HepaRG)	Reporter gene	Gain	RNA (mRNA)	Regulation of transcription factor activity	LTEA
UGT1a6	LTEA_HepaRG_UGT1A6_dn	Human	Liver (HepaRG)	Reporter gene	Loss	RNA (mRNA)	Regulation of transcription factor activity	LTEA
	LTEA_HepaRG_UGT1A6_up	Human	Liver (HepaRG)	Reporter gene	Gain	RNA (mRNA)	Regulation of transcription factor activity	LTEA
AhR	TOX21_AhR_LUC_Agonist	Human	Liver (HepG2)	Agonist	Gain	Protein (Receptor)	Regulation of transcription factor activity	TOX21
PXR	TOX21_PXR_Agonist	Human	Liver (HepG2)	Agonist	Gain	Protein (Receptor)	Regulation of transcription factor activity	TOX21
CAR	TOX21_CAR_Agonist	Human	Liver (HepG2)	Agonist	Gain	Protein (Receptor)	Regulation of transcription factor activity	TOX21
	TOX21_CAR_Antagonist	Human	Liver (HepG2)	Antagonist	Loss	Protein (Receptor)	Regulation of transcription factor activity	TOX21
CYP1a1	LTEA_HepaRG_CYP1A1_dn	Human	Liver (HepaRG)	Reporter gene	Loss	RNA (mRNA)	Regulation of transcription factor activity	LTEA
	LTEA_HepaRG_CYP1A1_up	Human	Liver (HepaRG)	Reporter gene	Gain	RNA (mRNA)	Regulation of transcription factor activity	LTEA
CYP1a2	LTEA_HepaRG_CYP1A2_dn	Human	Liver (HepaRG)	Reporter gene	Loss	RNA (mRNA)	Regulation of transcription factor activity	LTEA
	LTEA_HepaRG_CYP1A2_up	Human	Liver (HepaRG)	Reporter gene	Gain	RNA (mRNA)	Regulation of transcription factor activity	LTEA
THRSP	LTEA_HepaRG_THRSP_dn	Human	Liver (HepaRG)	Reporter gene	Loss	RNA (mRNA)	Regulation of transcription factor activity	LTEA
	LTEA_HepaRG_THRSP_up	Human	Liver (HepaRG)	Reporter gene	Gain	RNA (mRNA)	Regulation of transcription factor activity	LTEA

The assays were examined for the hit calls in the bioactivity data section (TOXCAST summary). Selection of the “Hit call” showed whether the test chemical was active or inactive. Following (17), a positive hit call is defined as “a biological perturbation having a maximum median response that exceed the cutoff defined for the assay and having data that can be curve-fit”. In addition, when the results were not clear, as was the case when a single endpoint was explored with 2 different assays with conflicting results, we added an “?” in our extraction files. The cytotoxicity limit for each chemical affected the number of active hit calls (18), however, we did not exclude chemicals that may be active only at high concentrations.

### 2.3 Study evaluation (risk of bias)

The internal validity of the selected studies was appraised with a risk of bias (RoB) assessment adapted from (19, 20; 21) and further developed for this systematic review. When a publication assayed several chemicals, we recorded one RoB entry for each chemical. The RoB tool consisted of a list of 18 questions with 4 response options based on (21) scores: ++ Definitely low risk of bias (dark green); + Probably low risk of bias (light green); ~ Probably high risk of bias (orange); ~~ Definitely high risk of bias (red). The tool was organized within Microsoft Excel and the template can be found in **Supplementary Material 3**.

Among the 18 questions, we included 8 key questions which had to achieve scores of “definitely” or “probably low risk” to rate a study as “high confidence”. These 8 key elements were:

1 and 2, Reliability and sensitivity of the analytical methods used for T4 and TSH quantification, respectively. As we did not have the resources to conduct a thorough review of this aspect, we scored articles as “~ Probably high risk of bias (orange)” or “~~ Definitely high risk of bias (red)” when i) details about the methodology or the assays were missing, ii) when we were unable to access details of the analytical measurements, for example, at the provider website, iii) when there was no mention of TH analytics, iv) when the assay used was developed for human serum without any further adaptation/validation for the rodent serum matrix. Accordingly, when the names of the assays employed were available, with technical details, we scored studies as “++ Definitely low risk of bias (dark green)” or “+ Probably low risk of bias (light green)”.

3. Exposure characterization, in terms of purity of the chemical, method of administration or measurement of real concentrations in the diet or drinking water. Studies with a comprehensive description of the exposure were classified as “++ Definitely low risk of bias (dark green)” or “+ Probably low risk of bias (light green)” when the study included a list of minimal information such as method of administration, concentrations, carrier solvent or duration of the exposure. When those details were missing or where we considered the information regarding the exposure as insufficient, the study was classified as “~ Probably high risk of bias (orange)”. Studies with direct evidence of inconsistent administrations were marked as “~~ Definitely high risk of bias (red)”.

4. Numbers of animals used per dose. Studies using 5 or more animals per experimental group were marked as “++ Definitely low risk of bias (dark green)”, while studies using fewer than 5 animals but with significant effects on TH/TSH levels were classified as “+ Probably low risk of bias (light green)”. Studies with insufficient information or direct evidence of using a lower number of animals per group were marked as “~ Probably high risk of bias (orange)” or “~~ Definitely high risk of bias (red)”, respectively.

5. Inclusion of a positive control for producing hormonal changes. Demonstration of effects with a positive control was taken as evidence of a responsive animal model. Accordingly, when a positive control was ineffective in terms of TH/TSH alterations, the study was ranked as “~~ Definitely high risk of bias (red)”. When a positive control showed an effect or was not included, but the treatment altered TH or TSH, the study was marked as “++ Definitely low risk of bias (dark green)” or “+ Probably low risk of bias (light green)”, respectively. If a positive control was not included and the treatments did not show effects, then, the study was classified as “~ Probably high risk of bias (orange)”.

6. Timing of sampling for TH/TSH measurements. When significant changes of TH/TSH levels were observed and the timing of sampling was in accordance with OECD/EPA test guidelines (2), we considered a study as “++ Definitely low risk of bias (dark green)”. If there were ambiguities in relation to compliance with test guideline recommendations (no specification of guidelines in the methodology), but significant effects were observed, we rated a study as “+ Probably low risk of bias (light green)”. Studies were marked as “~ Probably high risk of bias (orange)” when no significant changes were observed and there were ambiguities regarding adherence with test guidelines. “~~ Definitely high risk of bias (red)” studies with no changes and not adherence to guidelines (22).

7. Use of contemporaneous or historical controls as a comparator of hormonal changes. When a vehicle control was not employed, and data from historical controls were used as a comparator, the study was evaluated as “~~ Definitely high risk of bias (red)”, as identical test conditions cannot be guaranteed (See discussion in 23). When contemporaneous controls were used, we rated a study as “++ Definitely low risk of bias (dark green)”. The categories “+ Probably low risk of bias (light green)” and “~ Probably high risk of bias (orange)” were not used.

8. Use of soy-free animal feed. Soy-containing feeds can produce alterations in the hormonal levels and potentially mask the effects of EDCs (24) and can elicit goitrogenic effects in rats due to isoflavones (25–27). When the diet contained soy-derived components or alfalfa, the study was rated as “~~ Definitely high risk of bias (red)”. In cases where we were unable to trace the components of the diets, we scored the study as “+ Probably low risk of bias (light green)”. When the authors specified the diet as “soy-free”, then the study was ranked “++ Definitely low risk of bias (dark green)”.

To achieve an overall evaluation of each study, we adopted the decision rules detailed in EFSA (28) which encompasses 3 Tiers, as follows:

We placed studies in the highest confidence level (Tier 1, green) when all 8 key elements were scored ++ (Definitely low risk of bias) OR + (probably low risk of bias) AND no more than 1 question not addressing these key elements was scored ~ (probably high risk of bias) or ~~ (definitely high risk of bias).

A medium confidence level (Tier 2 orange) was used for all combinations not covered in Tier 1 or Tier 3.

The lowest confidence level (Tier 3 red) was reserved for studies where any one of the 8 key elements was rated ~ or ~~ OR more than 50% of the questions not addressing these key elements were scored ~ or ~~.

We piloted the RoB scheme by selecting a random sample of 10 studies which was scored independently by 3 different team members. Any conflict regarding RoB outcomes was reviewed and resolved after discussion between the team members.

## 3 Results

### 3.1 Study selection

**Figure 1:**
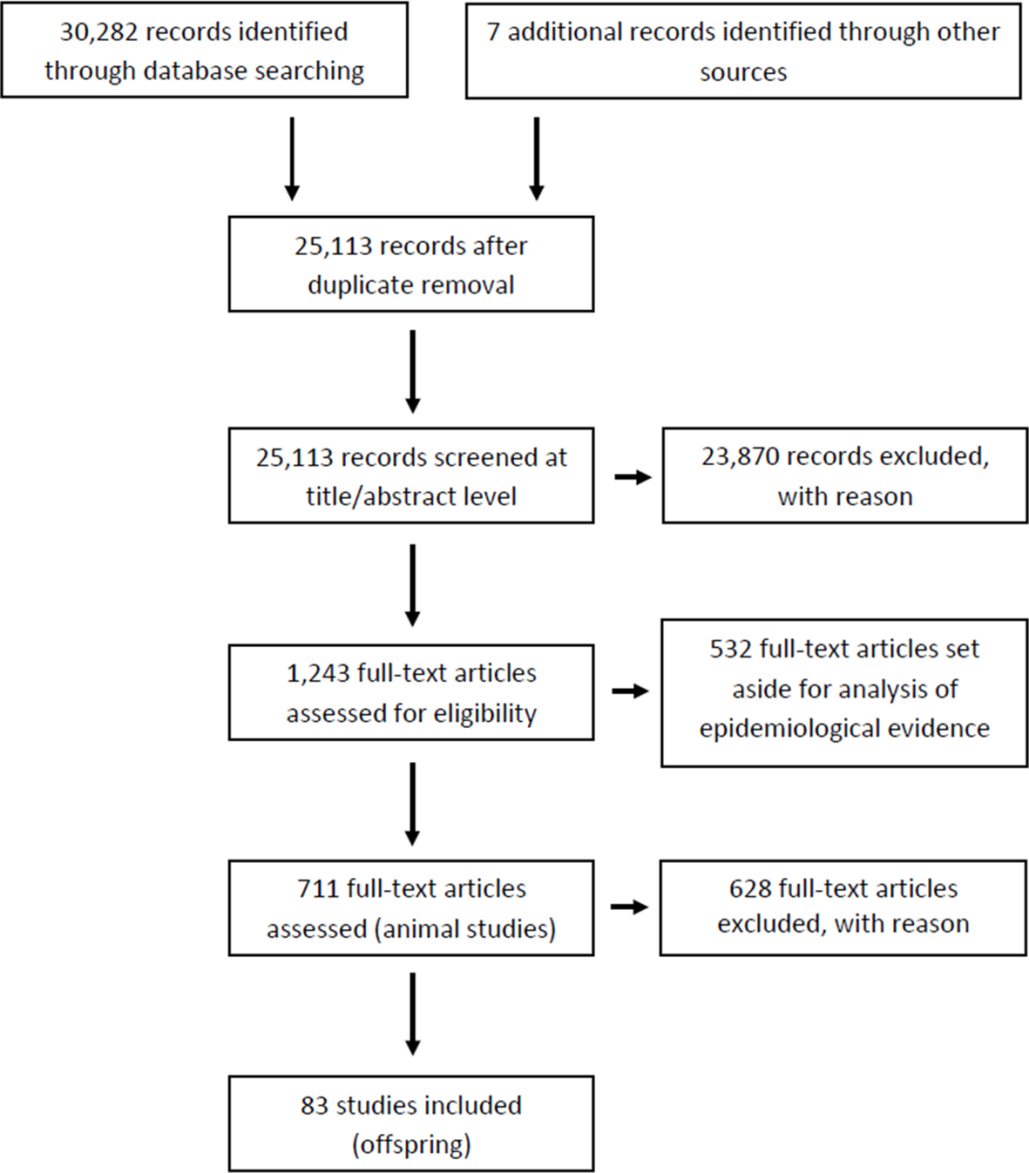
Prisma flow.

The outcome of our literature search and screening is shown in **Figure 1** (Prisma flow). We retrieved a total of 30,282 records from 3 different databases (Pubmed, Web of Science, Scopus) and 7 additional records through other sources (expert suggestions). After removal of duplicates, 25,113 records were manually screened for relevance (article titles and abstracts). Of these, 23,870 records were identified as not relevant or as not meeting our eligibility criteria, due to: i) exposure factors other than chemicals or their mixtures, ii) incomplete T4 and TSH data; iii) studies in systems other than *in vivo* experiments, or epidemiological studies. At this stage we also excluded all records that were not primary research papers.

The remaining 1,243 items were subjected to full-text screening based on our PECO criteria (see **Table 1**). Of those, 532 full-text records were set aside for another evidence mapping focusing on human epidemiology. Of the remaining 711 studies, 628 studies were excluded at this last step as they did not meet our PECO criteria due to i) adult rodent exposure (left aside for a follow-up evidence mapping for adult exposures); ii) non-gavage administration (e.g. injection, inhalation); iii) administration of the chemical outside the gestation and/or lactation period; iv) missing thyroid hormone data; v) use of animals with surgical modifications such as e.g. ovariectomy; vi) use of mixtures or lack of original data; vi) use of non-rodent species (See **Supplementary Material 4** for the list of articles for full-text screening with CADIMA reference numbers). Only one study using non-rodent animal models was found (CADIMA reference number in parentheses): ewe (2196). Due to the small number of animals in these non-rodent studies, and the difficulty of comparing the results with those from rodents, we excluded the study. This left a total of 83 records for in-depth analysis (**Supplementary Material 5**). Of those, a subset of 24 studies were experiments with only one dose group per test compound. These studies were taken out of the main body of the systematic review and dealt with separately. The remaining records were considered the most suitable for our purposes and were prioritized for data extraction and evaluation (**Supplementary Material 6** for multiple doses and **Supplementary Material 7** for studies with only one dose group).

### 3.2 Risk of bias evaluation

We evaluated the internal validity of eligible studies by conducting a risk-of-bias analysis (**Supplementary Material 8**). Because some studies reported the effects of multiple chemicals, we conducted the risk-of-bias analysis with a focus on each tested compound. Accordingly, the unit of evidence was the chemical and not the study.

As shown in **Table 3**, most “low confidence” units of evidence (Tier 3) did not meet our quality criteria for T4 or TSH analytics and quantification methods (key elements 1 and 2), mainly because of inadequate descriptions of method details, or because of the use of human immunoassays on rat sera without adaptation or validation.

**Table 3:** Risk of Bias ratings of each unit of evidence for all the questions involved in the risk-of-bias analysis for studies with two or more chemical doses.

Risk of Bias questions	++ Definitely low risk of bias	+ Probably low risk of bias	~ Probably high risk of bias	~~ Definitely high risk of bias
KEY ELEMENTS				
1. Were reliable and sensitive methods used for the T4 quantification?	1	27	31	0
2. Were reliable and sensitive methods used for the TSH quantification?	15	16	28	0
3. Was exposure sufficiently characterised?	33	26	0	0
4. Was the number of animals per dose group sufficient?	39	12	8	0
5. Was a positive control included?	1	56	2	0
6. Were measurements collected at a suitable timepoint when we compare control and treated groups?	7	50	2	0
7. Have the authors evaluated the hormonal changes in relation to contemporaneous or historical controls?	59	–	–	0
8. Was the diet free of phytoestrogens or/and goitrogens?	10	31	–	18
OTHER ELEMENTS				
Were animals randomly allocated to dose groups?	29	30	0	0
Was allocation to dose groups adequately concealed?	0	59	0	0
Were all experimental animals of similar age, strain, health status and source?	55	4	0	0
Were experimental conditions identical across study groups?	50	9	0	0
Were research personnel blinded to study groups?	0	59	0	0
Were outcome data complete without attrition or exclusion?	14	45	0	0
Were the factors that might influence the variability of TH measurements considered?	8	51	0	0
Have all study outcomes been reported?	23	35	0	1
Have funding sources and conflicts of interest been reported?	34	–	–	25
Were statistical methods reported in the study, appropriate and consistent?	14	44	1	0

Shadings are related to number of items, the higher the number, the darker the shading.

Another frequently encountered shortcoming was the use of soy-containing diets (key element 8). Furthermore, a few studies included insufficient numbers of animals per dose group. The quality criteria for all other key elements were generally met. **Supplementary Material 9** lists the risk-of-bias outcome for studies with only a single dose exposure group.

Of the 49 studies selected with more than 2 dose groups (representing 59 units of evidence), 12 units of evidence were scored as “definitely low” or “probably low risk” on all the key elements and for most of the remaining elements. We classed these studies as high-confidence studies (Tier 1). While no records were placed in Tier 2, our evaluation returned 47 units of evidence with a low confidence rating, placed in Tier 3 (**Table 4**). The same analysis was performed for studies with one dose group (**Supplementary Materials 9, 10**).

**Table 4:** Outcome of risk-of-bias assessment for each unit of evidence.

Tier	Units of evidence	Chemical (Cadima Reference Number)	Key Elements for RoB analysis (KE)							
			KE1	KE2	KE3	KE4	KE5	KE6	KE7	KE8
1	12	PCB 77 (1126)	+	++	++	+	+	+	++	+
		PCB 126 (1126)	+	++	++	+	+	+	++	+
		TCDD (1126)	+	++	++	+	+	+	++	+
		PFBS (2343)	+	+	+	++	+	+	++	+
		Amitrole (7478)	+	+	++	+	+	+	++	++
		Cyanamide (7478)	+	+	++	+	+	+	++	++
		2-Mercapt. (7478)	+	+	++	+	+	+	++	++
		DE-71 (930)	+	+	++	+	+	+	++	+
		PTU (930)	+	+	++	+	+	+	++	+
		PTU (AD 5)	+	++	+	++	+	+	++	+
		Triclosan (563)	+	+	+	++	+	+	++	+
		Perchlorate (AD 2)	+	++	+	++	+	+	++	+
2	0	–	–	–	–	–	–	–	–	–
3	47	BPA (255)	++	+	+	++	~	~	++	++
		BPA (2146)	+	~	+	++	+	+	++	+
		BPA (1933)	+	~	+	~	+	+	++	+
		TBBPA (2320)	~	~	++	++	+	+	++	++
		TBBPA (1456)	~	~	++	++	+	++	++	~~
		Aroclor 1254 (2275)	~	~	+	++	+	+	++	+
		Aroclor 1254 (3441)	~	~	++	++	+	+	++	+
		Aroclor 1254 (1947)	~	+	++	++	+	++	++	+
		PCB 153 (1885)	~	~	++	++	+	+	++	~~
		PCB 153 (2215)	~	~	++	++	+	+	++	~~
		4-OH-CB107 (3441)	~	~	++	++	+	+	++	+
		TCDD (738)	~	~	+	+	+	+	++	+
		TCDD (191)	~	~	+	++	+	+	++	+
		PFOS (2295)	+	++	++	++	+	+	++	~~
		PFOS (1765)	+	++	++	+	+	+	++	~~
		PFOS (1247)	~	~	+	+	+	+	++	~~
		PFHxS (2429)	~	+	+	++	+	+	++	++
		Chlorpyrifos (2118)	~	~	+	++	+	++	++	~~
		Ethylenthiourea (166)	~	~	+	++	+	+	++	~~
		Glyphosate (267)	~	+	+	++	+	+	++	+
		Vinclozolin (106)	~	~	++	++	+	++	++	+
		Vinclozolin (3257)	~	~	++	++	+	++	++	+
		PBDE-47 (6748)	~	++	++	++	+	+	++	~~
		DE-71 (1826)	+	++	++	++	+	+	++	~~
		DE-71 (5748)	+	++	++	+	+	++	++	~~
		DE-71 (6748)	~	++	++	++	+	+	++	~~
		DE-71 (2218)	~	~	++	++	+	+	++	~~
		DBDE (14143)	~	~	+	++	+	+	++	++
		HBCD (2320)	~	~	++	++	+	+	++	++
		DE-209 (1172)	~	~	++	++	+	+	++	+
		Perchlorate (18235)	+	+	+	++	+	+	++	~~
		Indomethacin (27596)	~	~	+	++	+	+	++	+
		DEHP (527)	~	~	+	++	+	+	++	+
		DHP (758)	+	+	++	~	+	+	++	+
		DHP (2863)	+	+	++	~	+	+	++	+
		DCHP (758)	+	+	++	~	+	+	++	+
		DCHP (2863)	+	+	++	~	+	+	++	+
		Triclosan (304)	~	~	++	++	+	+	++	+
		Triclosan (1657)	+	++	++	++	+	+	++	~~
		4-Nonylphenol (4293)	~	~	+	++	+	+	++	~~
		4-Nonylphenol (299)	~	~	+	++	++	+	++	+
		Genistein (14584)	~	~	+	~	~	~	++	++
		Selenium (22710)	~	~	+	++	+	+	++	+
		PTU (AD 3)	+	++	+	~	+	+	++	+
		PTU (AD 4)	+	++	+	~	+	+	++	+
		PTU (AD 6)	~	~	+	++	+	+	++	~~
		PTU (AD 7)	+	++	++	++	+	++	++	~~

Shadings are related to number of items, the higher the number, the darker the shading.

### 3.3 Characteristics of eligible studies and T4 - TSH response patterns

Most studies were conducted in the rat; only 3 out of 83 studies used mice (Cadima reference numbers 563 [triclosan], 2343 [PFBS] and 4842 [mancozeb, imidacloprid]). All the test chemicals were administered orally: by diet, via drinking water or intragastrical, during gestation and/or lactation. Most studies covered the period when the thyroid gland develops during gestation (GD9 to GD17 in the rat) and when it becomes functional (GD17 to GD21).

Only a few studies exposed the animals before pregnancy, prior to mating or during mating (Cadima reference numbers 1456, 1247, 2118, 29594, 106, 3257, 4293). We also included 4 articles where free, unbound T4 (fT4) was measured instead of T4 (Cadima reference numbers 299, 1172, 2261 and 11193). Most of the studies extracted the T4 and TSH from serum as recommended in ATA guidelines (29); only 17 studies used plasma to quantify the hormones.

The levels of T4, TSH and, when available, fT4, were reported as an increase or decrease (statistically significant changes) or no change in relation to a control group (no test compound exposure). We calculated the percentage of such changes in relation to control groups for all the test compounds (see **Supplementary Material 6** for further details for multiple doses and **Supplementary Material 7** for single-dose studies).

### 3.4 Types of altered T4 – TSH levels

**Figure 2:**
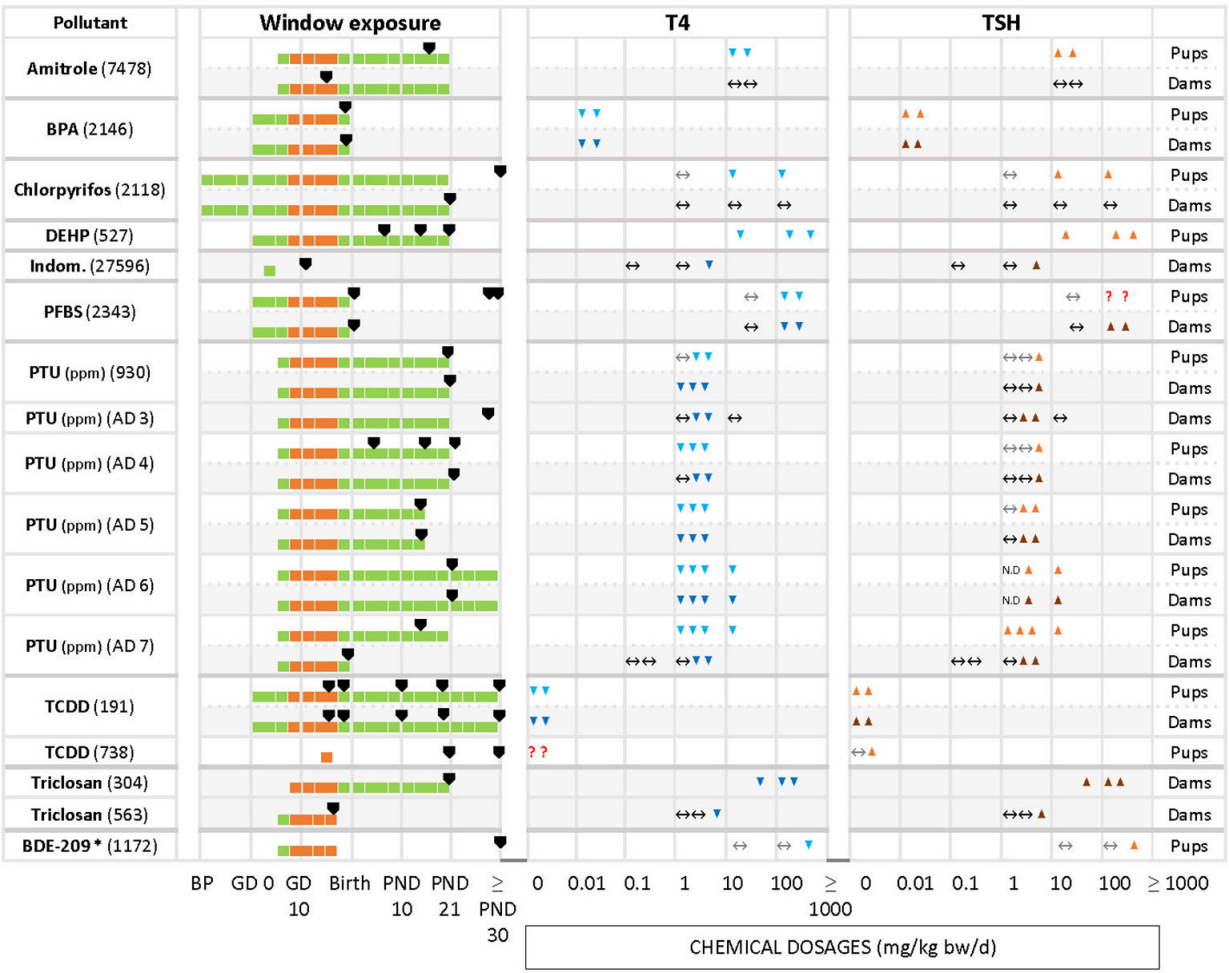
Summary of T4 – TSH response patterns with chemicals producing T4 decrements and TSH increases in dams and pups. Shown are records for test compounds with corresponding Cadima reference numbers (see **Supplementary Material 5**). Light grey cells show responses in dams, white cells in pups. Windows of exposure were labelled BP (pre-mating/mating periods, before pregnancy), GD (gestational days, GD 0 to birth) and PND (postnatal days from 10 to 30 or after 30) and green horizontal bars depict the duration of the exposure to pregnant dams. The period of thyroid development (GD 9 – GD 17, 30) is shown as an orange bar. Black arrowheads “▼” indicate the timing of blood sampling for T4 and TSH measurements. Blue downward arrowheads stand for T4 decrements, light blue “▼” in pups and dark blue “▼” in dams; orange upwards arrowheads show increases of T4 or TSH, light orange “▲”in pups, dark orange “▲” in dams. The absence of change in hormonal levels is represented by “↔”. Where varying responses occurred, we used “**?**”. Most of the studies reported dosages of test chemicals as mg/kg bw/d, except for PTU administered via the drinking water where doses were expressed as parts per million (ppm) (indicated next to the chemical name). The asterisk (*) indicates studies where FT4 was measured instead of T4. When the exposure (green horizontal bars) reached “≥ PND 30”, this indicates that the dosing period was prolonged until or beyond the PND 30. When the sampling day was placed on the “≥ PND 30”, this indicates that the sampling took place on the 30 PND or after this day (for more details, see the **Supplementary Materials 5–7**).

**Figure 3:**
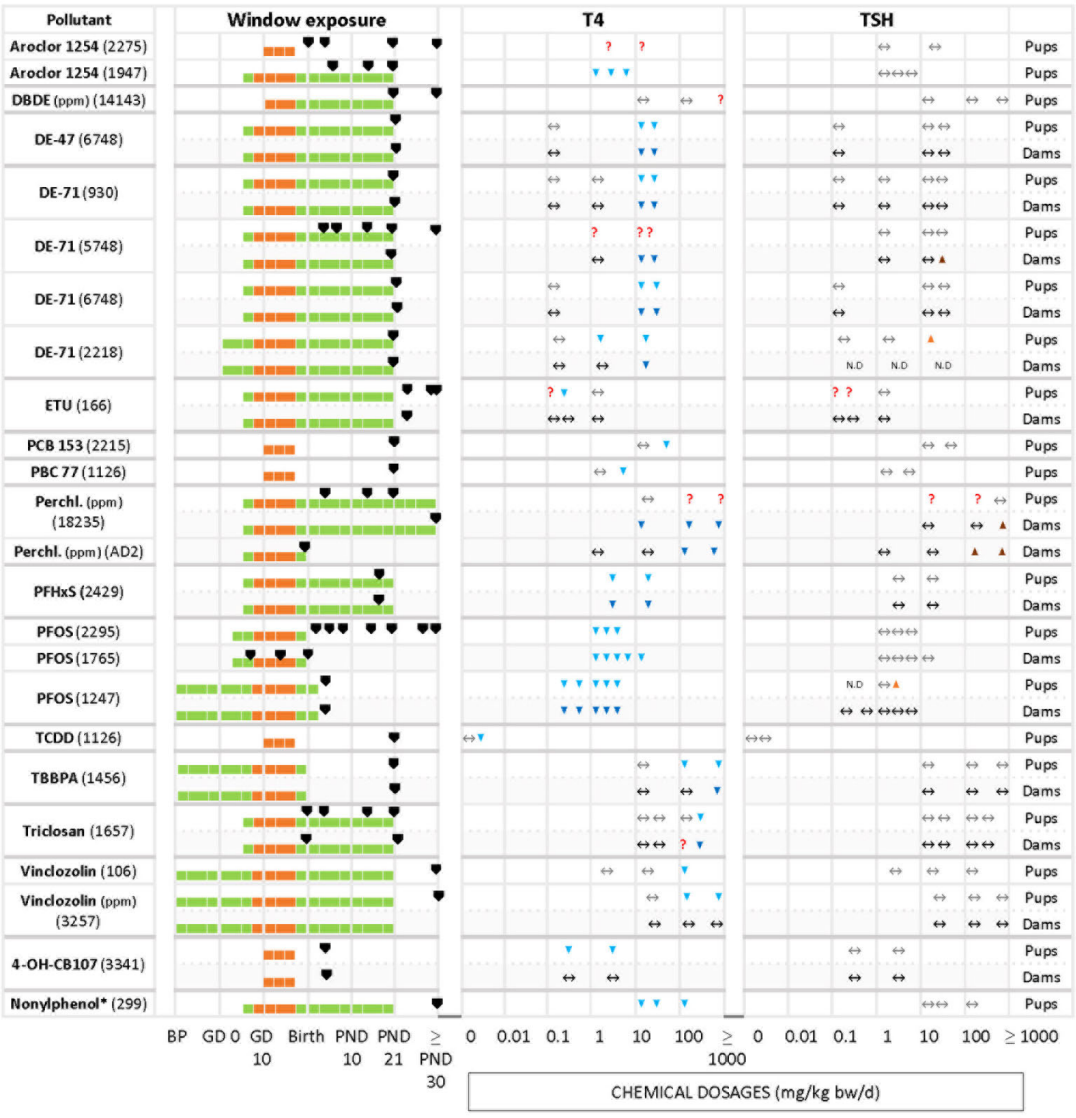
Summary of T4 – TSH response patterns with chemicals producing T4 serum decrements with no TSH changes in dams and pups. Symbols and shading as in **Figure 2**.

**Figure 4:**
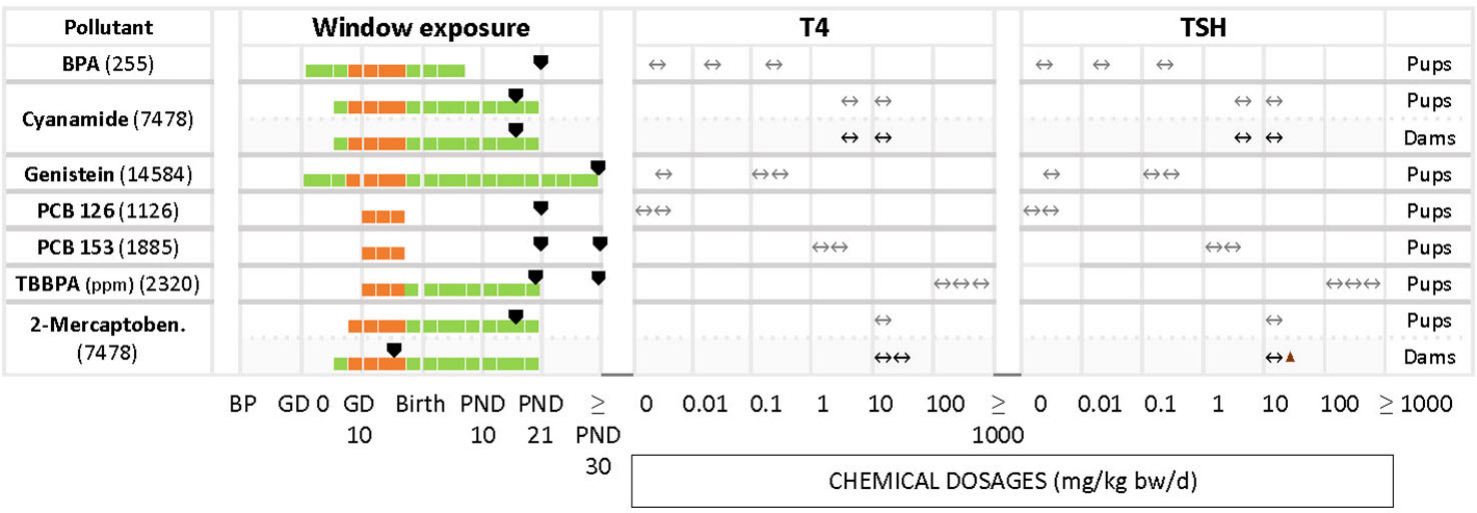
Summary of studies where T4 - TSH changes were not observed in dams and pups at various dosages after gestational and perinatal exposures to test chemicals. List of studies showing as main response the unaffected levels of T4 and TSH. Symbols and shading as in **Figure 2**.

**Figure 5:**
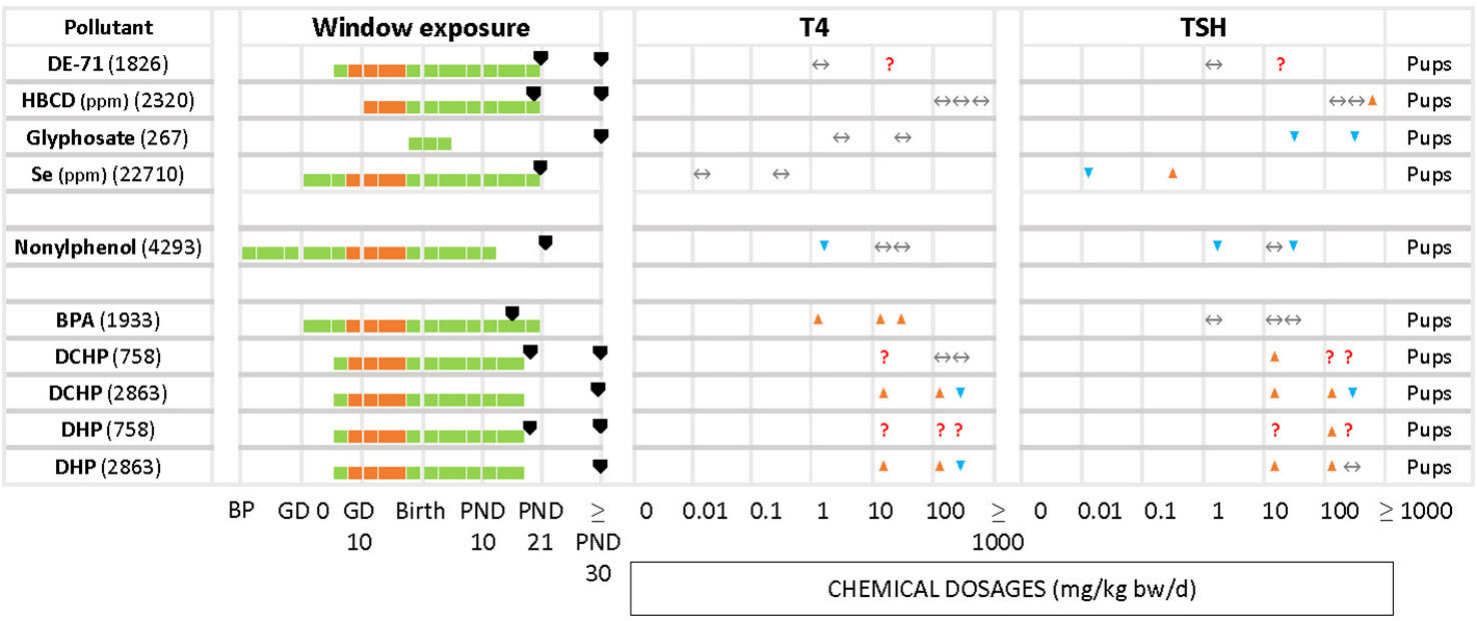
Summary of T4/TSH response patterns in dams and pups for chemicals producing inconsistent effects. Symbols and shading as in **Figure 2**. (See **Supplementary Material 10** for single-dose studies).

We grouped chemicals according to T4 - TSH response patterns observed in dams and pups, as follows: Suppressions of serum T4 levels followed by increases in TSH (**Figure 2**), decreases of serum T4, with no changes in TSH (**Figure 3**), and mixed patterns, with either unchanged or increased T4 and varying TSH changes (**Figures 4**, **5**). **Supplementary Material 11** lists similar patterns but for studies where only one dose group was used.

#### 3.4.1 T4 serum decrements accompanied by TSH increases

Decreased serum T4 with increased TSH is one of the two response patterns which stands out as the most frequently observed (**Figure 2**). Chemicals such as amitrole (7478 in pups), BPA (2146 in dams and pups), DEHP (527 in pups), PTU (AD6 in dams and pups, AD 7 in pups), TCDD (191 in dams and pups) and triclosan (304 in dams) showed significant changes for T4/TSH at all doses tested. With PTU (AD4 in pups, AD5 in dams and pups, AD6 in dams and pups, AD7 in pups), most of the exposed groups showed significantly reduced serum T4 followed by an increase in TSH (although this was not observed when the PTU doses were low). Some other studies, such as with chlorpyrifos in pups (2118), indomethacin in dams (27596), PFBS (2343 in dams and pups), PTU (930, AD4 and AD5 in dams and pups, AD3 in dams), triclosan in dams (563) and BDE-209 in pups (1172) only reported significant changes for T4 and/or TSH at higher dosages. We noticed that some compounds, such as amitrole (7478), chlorpyrifos (2118) and PTU (930, AD4, AD7) produced different responses in dams and pups (see next section). TCDD (738) administered as single oral dose (200, 800 ng/kg bw) at GD 15 did not show a clear trend for T4 in pups. In this case, T4 was measured at 2 different time points for male and female, PND 21 and PND 49. While TCDD decreased the levels of T4 in both sexes at PND 21, T4 was exclusively augmented in males at PND 49 with the highest concentration. Another example is PFBS (2343) for pups: although there was an increase for TSH with 200 and 500 mg/kg bw/d, significant differences were only reported for PND 30, and not at PND 1 or PND 60.

#### 3.4.2 T4 serum decrements without accompanying TSH increases

The other frequently observed response pattern is characterized by T4 serum decrements without corresponding increases in TSH levels (**Figure 3**). This applies to Aroclor 1254 (1947 in pups), PFHxS (2429 in dams and pups), PFOS (2295 in pups, 1765 and 1247 in dams), 4-OH-CB107 (3341 in pups) and nonylphenol (299 in pups). In some studies T4 decrements were only observed at higher doses (relative to the dose range tested in that particular study), such as with DBDE (14143 in pups), DE-47 (6748 in pups and dams), DE-71 (930, 6748 and 2218 in dams and pups, 5748 in dams), PCB 153 (2215 in pups), PCB 77 (1126 in pups), TCDD (1126 in pups), TBBPA (1456 in pups and dams), triclosan (1657 in pups and dams), and vinclozolin (106 and 3257 in pups).

ETU (166) did not show a clear response pattern in offspring. In female pups, there were decrements of serum T4 at an intermediate dose at three sampling times (PND 23, 42, 75), while in male offspring the levels of T4 remained unchanged (PND 42, and with the lowest and highest dosages). TSH levels did not show a clear response pattern, with an increase at 0.1 mg/kg bw/d in PND 23 males and PND 75 females. A few chemicals produced an increase of TSH solely at higher doses. Examples are DE-71 (5748 for dams, 2218 for pups), perchlorate (18235 and AD2 in dams) and PFOS (1247 for pups).

Differences in the timing of serum T4 and TSH measurements may have produced these varying response patterns. Such cases are highlighted by the “?” symbols in **Figure 2B**.

#### 3.4.3 Other response patterns

In some studies where T4 and TSH were measured the test chemicals produced no alterations, although distinct changes had been reported by others (**Figure 4**). Examples include cyanamide for pups and dams (7458), BPA (255), genistein (14584), PCB 126 (1126), PCB 153 (1885) and TBBPA (2320) in pups.

Less frequently seen patterns include unchanged T4 levels with increased or decreased TSH (**Figure 5**), as in the case of HBCD (2320) for the highest concentration, glyphosate (267). Increased serum T4 with unchanged TSH was observed with BPA (1933). In the study of DE-71 (1826), T4 and TSH were measured at PND 21 and PND 60 for 2 different concentrations, 1.7 and 30.6 mg/kg bw/d. However, T4 was significantly decreased only at PND 21 with the highest concentration tested. TSH was increased at the highest dose, at PND 60. Nonylphenol (4293) yielded significant declines in TSH and T4 at the lowest tested dose (2 mg/kg bw/d), observed in both male and female offspring. Additionally, females showed a significant decrease of TSH at the highest dose (50 mg/kg bw/d). There were 2 studies with pregnant rats exposed from GD 6 to GD 19 to 3 different doses (20, 100, 500 mg/kg bw/d) of phthalates, DCHP and DHP (748 and 2863). In study 748, the hormones were measured at PND 20 and PND 32, while study 2863 measured at PND 90 for both male and female offspring. However, the results showed varied responses for TSH and T4 for both chemicals.

Percentage changes of T4 and TSH were calculated with respect to the control (see **Supplementary Materials 5–7**). Significant decrements of T4 (pups) for the first group of chemicals (T4 down, TSH up) varied from -8% (DEPH, 527) to -92% (PTU, AD7) with increments in TSH from 16% (PFBS, 2343) to 750% (PTU, AD4). For the dams, the percentage of change in T4 ranged from -12% (PTU, AD6) to -90% (PTU, AD5), and from 10% (triclosan, 304) to 2843% (PTU, AD5) for TSH.

In the second group of chemicals (T4 down, TSH unmodified), the percentage of T4 in pups varied between -8% (PFOS, 2295) to -100% (PFOS, 1247).

### 3.5 Consistency of response patterns across studies

Certain chemicals showed variable T4 – TSH response patterns in different studies (**Table 5**, **Supplementary Material 7** for studies with only one dose group). In many cases, the discrepancies can be explained in terms of differences in the doses administered (e.g. Aroclor 1254, PCB 153, TCDD, DE-71, PTU), treatment duration (TCDD) and hormone measurement time points (Aroclor 1254, DE-71).

**Table 5:** Overview of the test compounds repeatedly assessed in different studies.

						Pups		Dams	
Acronym	CADIMA ref. number	Start of dosing	Outcome assessment	Treatment duration	Exposure concentration	T4	TSH	T4	TSH
BPA	255	GD 0	PND 21	Gest. – PND 9	4, 40, 400 µg/kg bw/d	↔	↔	**ND**	**ND**
	2146	GD 1	GD 20	GD 1 - GD 20	20, 40 µg/kg bw/d	**▼**	**▲**	**▼**	**▲**
	1933	Gestation	PND 15	Gest. – lact.	1, 10, 50 mg/kg bw/d	**▲**	↔	**ND**	**ND**
TBBPA	2320	GD 10	PND 20	GD 10 - PND 20	100, 1000, 10000 ppm	↔	↔	**ND**	**ND**
			PND 77		100, 1000, 10000 ppm	↔	↔	**ND**	**ND**
	1456	Premating	PND 21	Premating, mating, gestation	10 mg/kg bw/d	↔	↔	↔	↔
					100 mg/kg bw/d	**▼**	↔	**▼/**↔	↔
					1000 mg/kg bw/d	**▼**	↔	**▼**	↔
Aroclor 1254	2275	GD 10	GD 20	GD 10 - GD 16	5, 25 mg/kg bw/d	**▼**	↔	**ND**	**ND**
			PND 4		5, 25 mg/kg bw/d	**▼**	↔	**ND**	**ND**
			PND 21		5 mg/kg bw/d	↔	↔	**ND**	**ND**
					25 mg/kg bw/d	**▼**	↔	**ND**	**ND**
			PND 90		5 mg/kg bw/d	**▲/**↔	↔	**ND**	**ND**
					25 mg/kg bw/d	↔	↔	**ND**	**ND**
	1947	GD 6	PND 7	GD 6 - PND 21	1 mg/kg bw/d	**▼**	↔	**ND**	**ND**
					4, 8 mg/kg bw/d	**▼**	**ND**	**ND**	**ND**
			PND 14		1, 4, 8 mg/kg bw/d	**▼**	↔	**ND**	**ND**
			PND 21		1, 4 mg/kg bw/d	**▼**	↔	**ND**	**ND**
					8 mg/kg bw/d	**ND**	↔	**ND**	**ND**
PCB 153	1885	GD 10	3, 9 weeks	GD 10 - GD 16	1, 4 mg/kg bw/d	↔	↔	**ND**	**ND**
	2215	GD10	PND 21	GD 10 - GD 16	16 mg/kg bw/d	↔	↔	**ND**	**ND**
					64 mg/kg bw/d	**▼**	↔	**ND**	**ND**
TCDD	738	GD 15	PND 21	1 day	200 ng/kg bw	**▼**	↔	**ND**	**ND**
					800 ng/kg bw	**▼**	**▲**	**ND**	**ND**
			PND 49		200 ng/kg bw	↔	↔	**ND**	**ND**
					800 ng/kg bw	**▲/**↔	**▲**	**ND**	**ND**
	1126	GD 10	PND 21	GD 10 - GD 16	0.025 µg/kg bw/d	↔	↔	**ND**	**ND**
					0.1 µg/kg bw/d	**▼**	↔	**ND**	**ND**
	191	GD 1	GD16, 19	GD 1 - PND 30	0.2, 0.4 µg/kg bw/d	**▼**	**▲**	**▼**	**▲**
			PND 10, 20, 30		0.2, 0.4 µg/kg bw/d	**▼**	**▲**	**▼**	**▲**
PFOS	2295	GD 2	PND 2, 5, 9, 15, 21, 28, 35	GD 2 - GD 21	1, 2, 3 mg/kg bw/d	**▼**	↔	**ND**	**ND**
	1765	GD 2	GD 7, 14, 21	GD 2 - GD 20	1,2, 3, 5, 10 mg/kg bw/d	**ND**	**ND**	**▼**	↔
	1247	42 days premating	PND 5	42 days premating - PND 4	0.4, 0.8,1 mg/kg bw/d	**▼**	**ND**	**▼**	↔
					1.2 mg/kg bw/d	**▼**	↔	**▼**	↔
					1.6 mg/kg bw/d	**▼**	**▲**	**▼**	↔
					2 mg/kg bw/d	**ND**	**ND**	**▼**	↔
Vinclozolin	106	Premating	PND 71	Premating - PND 21	4, 20 mg/kg bw/d	↔	↔	**ND**	**ND**
					100 mg/kg bw/d	**▼**	↔	**ND**	**ND**
	3257	Premating	Dams: ? Pups: 21–23 weeks	Premating to weaning	40 ppm	↔	↔	↔	↔
					200 ppm	**▼/**↔	↔	**▼**	↔
					1000 ppm	**▼**	↔	**▼**	↔
DE-71	1826	GD 6	PND 21	GD 6 - PND 21	1.7mg/kg bw/d	↔	↔	**ND**	**ND**
					30.6 mg/kg bw/d	**▼**	**▼**	**ND**	**ND**
			PND 60		1.7mg/kg bw/d	↔	↔	**ND**	**ND**
					30.6 mg/kg bw/d	↔	**▲**	**ND**	**ND**
	930	GD 6	PND 21	GD 6 - PND 21	0.1, 1 mg/kg bw/d	↔	↔	↔	↔
					10, 30 mg/kg bw/d	**▼**	↔	**▼**	↔
	5748	GD 6	PND 4	GD 6 - PND 21	1.7, 10.2, 30.6 mg/kg bw/d	↔	↔	**ND**	**ND**
			PND 7		1.7 mg/kg bw/d	↔	↔	**ND**	**ND**
					10.2, 30.6 mg/kg bw/d	**▼/**↔	↔	**ND**	**ND**
			PND 14		1.7 mg/kg bw/d	↔	↔	**ND**	**ND**
					10.2, 30.6 mg/kg bw/d	**▼**	↔	**ND**	**ND**
			PND 21		1.7 mg/kg bw/d	↔	↔	↔	↔
					10.2 mg/kg bw/d	**▼**	↔	**▼**	↔
					30.6 mg/kg bw/d	**▼**	↔	**▼**	**▲**
			PND 60		1.7 mg/kg bw/d	↔	**▼/**↔	**ND**	**ND**
					10.2, 30.6 mg/kg bw/d	**▲/**↔	**▼/**↔	**ND**	**ND**
	6748	GD 6	PND 22	GD 6 - PND 21	0.1mg/kg bw/d	↔	↔	↔	↔
					15, 30 mg/kg bw/d	**▼**	↔	**▼**	↔
	2218	GD 1	PND 21	GD 1 - PND 21	0.3 mg/kg bw/d	↔	↔	↔	**ND**
					3 mg/kg bw/d	**▼**	↔	↔	**ND**
					30 mg/kg bw/d	**▼**	**▲**	**▼**	**ND**
PTU	930	GD 6	PND 27	GD 6 - PND 21	1 ppm	↔	↔	**▼**	↔
					2 ppm	**▼**	↔	**▼**	↔
					3 ppm	**▼**	**▲**	**▼**	**▲**
	AD 3	GD 6	PND 22	GD 6 - PND 21	1, 10 ppm	**ND**	**ND**	↔	↔
					2, 3 ppm	**ND**	**ND**	**▼**	**▲**
	AD 4	GD 6	PND 4	GD 6 - PND 21	1, 2 ppm	↔	↔	**ND**	**ND**
					3 ppm	↔	**▲**	**ND**	**ND**
			PND 15		1 ppm	**▼**	↔	**ND**	**ND**
					2,3 ppm	**▼**	**▲**	**ND**	**ND**
			PND 22		1 ppm	**▼**	↔	↔	↔
					2 ppm	**▼**	↔	**▼**	↔
					3 ppm	**▼**	**▲**	**▼**	**▲**
	AD 5	GD 6	PND 14	GD 6 - PND 14	1 ppm	**▼**	↔	**▼**	**–**
					2,3 ppm	**▼**	**▲**	**▼**	**▲**
			PND 16	GD 6 - PND 16	10 ppm	**ND**	**▲**	**▼**	**▲**
	AD 6	GD 6	PND 21 - 23	GD 6 - PND 30	1, 2 ppm	**▼**	**ND**	**▼**	**ND**
					3, 10 ppm	**▼**	**▲**	**▼**	**▲**
	AD 7	GD 6	GD 20 (dams) PND 14 (pups)	GD 6 – GD 20 (dams) GD 6 - PND 21 (pups)	0.1, 0.5 ppm	**ND**	**ND**	↔	↔
					1 ppm	**▼**	**▲**	↔	↔
					2, 3 ppm	**▼**	**▲**	**▼**	**▲**
					10 ppm	**▼**	**▲**	**ND**	**ND**
DHP	758	GD 6	PND 20	13 days	20 mg/kg/day	**▲**	↔	**ND**	**ND**
					100 mg/kg/day	**▲**	**▲**	**ND**	**ND**
					500 mg/kg/day	**▲**	**▲/**↔	**ND**	**ND**
			PND 32		20 mg/kg/day	↔	**▼/**↔	**ND**	**ND**
					100 mg/kg/day	↔	**▲/**↔	**ND**	**ND**
					500 mg/kg/day	↔	↔	**ND**	**ND**
	2863	GD 6	PND 90	13 days	20 mg/kg bw/d	**▲**	**▼/**↔	**ND**	**ND**
					100 mg/kg bw/d	**▲**	**▼**	**ND**	**ND**
					500 mg/kg bw/d	**▼/**↔	↔	**ND**	**ND**
DCHP	758	GD 6	PND 20	13 days	20 mg/kg/day	**▲/**↔	**▲/**↔	**ND**	**ND**
					100, 500 mg/kg/day	↔	**▲/**↔	**ND**	**ND**
			PND 32		20 mg/kg/day	↔	**▲/**↔	**ND**	**ND**
					100, 500 mg/kg/day	↔	↔	**ND**	**ND**
	2863	GD 6	PND 90	13 days	20 mg/kg bw/d	**▲/**↔	**▲/**↔	**ND**	**ND**
					100 mg/kg bw/d	**▲**	**▲**	**ND**	**ND**
					500 mg/kg bw/d	**▼/**↔	**▼/**↔	**ND**	**ND**
Triclosan	304	GD 8	PND 21	GD 8 - PND 21	75, 150, 300 mg/kg bw/d	**ND**	**ND**	**▼**	**▲**
	563 (mice)	GD 6	GD 17	GD 6 - 18	1, 4 mg/kg bw/d	**ND**	**ND**	↔	↔
					8 mg/kg bw/d	**ND**	**ND**	**▼**	**▲**
	1657	GD 6	DG 20	GD 6 - PND 21	10, 20, 100 mg/kg bw/d	↔	↔	↔	↔
					300 mg/kg bw/d	**▼**	↔	**▼**	↔
			PND 4		10, 20, 100 mg/kg bw/d	**–**	↔	**ND**	**ND**
					300 mg/kg bw/d	**▼**	↔	**ND**	**ND**
			PND 14		10, 20, 100, 300 mg/kg bw/d	↔	↔	**ND**	**ND**
			PND 22		10, 20 mg/kg bw/d	↔	↔	↔	↔
					100, 300 mg/kg bw/d	↔	↔	**▼**	↔
Perchlorate	18235	GD 6	PND 4	GD 6 - PND 30	30, 300, 1000 ppm	↔	↔	**ND**	**ND**
			PND 14		30, 300 ppm	↔	**▼**	**ND**	**ND**
					1000 ppm	↔	↔	**ND**	**ND**
			PND 21 (pups), PND 30 (dams)		30 ppm	↔	↔	**▼**	↔
					300 ppm	**▼**	↔	**▼**	↔
					1000 ppm	**▼**	↔	**▼**	**▲**
	AD 2	GD 6	GD 20 (dams)	GD 6 - GD 20	1, 30 ppm	**ND**	**ND**	↔	↔
					300, 1000 ppm	**ND**	**ND**	**▼**	**▼**

Shown are names or acronyms of the test compounds, Cadima reference number, age at beginning of dosing, age at outcome assessment, duration of the treatment, chemical dosages, chemical names, and responses for T4/TSH in dams and pups. Blue arrowheads “▼” indicate decrements and orange arrowheads “▲” increases. No significant changes in hormonal levels are represented by “↔”. Absence of data is denoted as ND (no data).

However, the divergent patterns seen with BPA and TBBPA are hard to explain. While one study of BPA (255) did not produce any changes in T4 or TSH in pups, T4 decrements with accompanying TSH rises were observed in another BPA experiment (2146), despite a shorter treatment period. A third BPA study (1933), in contrast, found T4 increases without changes in TSH, in line with the observed *in vitro* TR antagonist properties, although lower doses than in the first BPA study (255) were used.

In the case of TBBPA, both studies used comparable doses, covered the period of thyroid gland development in gestation and conducted hormone measurements at similar time points, yet one (2320) did not find T4 or TSH changes in pups, while the other (1456) observed T4 decrements without changes in TSH. There were differences in the timing of dosing. Study 2320 started dosing at GD10 and study 1456 during the premating period.

### 3.6 Concordance of response patterns in dams and pups

By considering studies where TH and TSH were measured in dams and pups, we assessed whether the direction of hormonal changes was similar in dams and pups.

The direction of changes in dams and pups was consistent in studies of BPA (2146), TCDD (191) and PTU (930, AD 4 – 7), with decreased T4 and increased TSH, and BDE-47 (6748), DE-71 (930, 5748, 6748, 2218), perchlorate (18235) or PFHxS (2429) with decrements in T4 and unchanged TSH.

However, other chemicals showed a lack of concordance between dams and pups. Amitrole (7478), Chlorpyrifos (2118), ETU (166), 4-OH-CB107 (3441) were not able to alter the TH in dams but evoked TH changes in the pups reducing T4 with different effects on TSH (see **Table 6**).

**Table 6:** Overview of the T4/TSH levels simultaneously assessed in dams and pups.

						Pups		Dams	
Acronym	CADIMA ref. number	Start of dosing	Outcome assessment	Treatment duration	Exposure concentration	T4	TSH	T4	TSH
BPA	2146	GD 1	GD 20	GD 1 - GD 20	20, 40 ug/kg bw/d	**▼**	**▲**	**▼**	**▲**
TCDD	191	GD 1	GD16, 19	GD 1 - PND 30	0.2, 0.4 ug/kd bw/d	**▼**	**▲**	**▼**	**▲**
			PND 10, 20, 30		0.2, 0.4 ug/kd bw/d	**▼**	**▲**	**▼**	**▲**
Amitrole	7478	GD 7	GD 15 (dams) PND 16 (pups)	GD 7 - PND 22	25, 50 mg/kg bw/d	**▼**	**▲**	**↔**	**↔**
PFBS (mice)	2343	GD 1	PND 1	GD 1 - GD 20	50 mg/kg bw/d	**↔**	**↔**	**↔**	**↔**
					200, 500 mg/kg bw/d	**▼**	**▲/↔**	**▼**	**▲**
Chlorpyrifos	2118	Premating	PND21 (dams), PND 91 (pups)	Premat., mat., gest., lact.	1 mg/kg bw/d	**↔**	**↔**	**↔**	**↔**
					10 mg/kg bw/d	**▼/↔**	**▲/↔**	**↔**	**↔**
					100 mg/kg bw/d	**▼**	**▲**	**↔**	**↔**
PTU	930	GD 6	PND 21	GD 6 - PND 21	1 ppm	**↔**	**↔**	**▼**	**↔**
					2 ppm	**▼**	**↔**	**▼**	**↔**
					3 ppm	**▼**	**▲**	**▼**	**▲**
	AD 4	GD 6	PND 22	GD 6 - PND 21	1 ppm	**▼**	**↔**	**↔**	**↔**
					2 ppm	**▼**	**↔**	**▼**	**↔**
					3 ppm	**▼**	**▲**	**▼**	**▲**
	AD 5	GD 6	PND 14	GD 6 - PND 14	1 ppm	**▼**	**–**	**▼**	**–**
					2, 3 ppm	**▼**	**▲**	**▼**	**▲**
		GD 6	PND 16	GD 6 - PND 16	10 ppm	**ND**	**▲**	**▼**	**▲**
	AD 6	GD 6	PND 21 - 23	GD 6 - PND 30	1, 2 ppm	**▼**	**ND**	**▼**	**ND**
					3, 10 ppm	**▼**	**▲**	**▼**	**▲**
	AD 7	GD 6	PND 14 (pups), GD 20 (dams)	GD 6 – GD 20 (dams) GD 6 - PND 21 (pups)	1 ppm	**▼**	**▲**	**↔**	**↔**
					2, 3 ppm	**▼**	**▲**	**▼**	**▲**
TBBPA	1456	Premating	PND 21	Premat., mat., gest.	10 mg/kg bw/d	**↔**	**↔**	**↔**	**↔**
					100 mg/kg bw/d	**▼**	**↔**	**▼/**↔	**↔**
					1000 mg/kg bw/d	**▼**	**↔**	**▼**	**↔**
4-OH-CB	3441	GD 10	PND 4	GD 10 - GD 16	0.5, 5 mg/kg bw/d	**▼**	**↔**	**↔**	**↔**
PFOS	1247	42 days premating	PND 5	42 days premating - PND 4	0.4, 0.8, 1 mg/kg bw/d	**▼**	**ND**	**▼**	**↔**
					1.2 mg/kg bw/d	**▼**	**↔**	**▼**	**↔**
					1.6 mg/kg bw/d	**▼**	**▲**	**▼**	**↔**
					2 mg/kg bw/d	**ND**	**ND**	**▼**	**↔**
PFHxS	2429	GD 7	PND 16/17	GD 7 - PND 21	5, 25 mg/kg bw/d	**▼**	**↔**	**▼**	**↔**
ETU	166	GD 7	PND 23	Gestation, lactation	0.1 mg/kg bw/d	**▼/↔**	**▲/↔**	**↔**	**↔**
					0.3 mg/kg bw/d	**▼/↔**	**▲/↔**	**↔**	**↔**
					1 mg/kg bw/d	**↔**	**↔**	**↔**	**↔**
Vinclozolin	3257	Premating	Dams: ? Pups: 21–23 weeks	Premating to weaning	40 ppm	**↔**	**↔**	**↔**	**↔**
					200 ppm	**▼/↔**	**↔**	**▼**	**↔**
					1000 ppm	**▼**	**↔**	**▼**	**↔**
PBDE-47	6748	GD 6	PND 22	GD 6 - PND 21	0.1 mg/kg bw/d	**↔**	**↔**	**↔**	**↔**
					15, 50 mg/kg bw/d	**▼**	**↔**	**▼**	**↔**
DE-71	930	GD 6	PND 21	GD 6 - PND 21	0.1, 1 mg/kg bw/d	**↔**	**↔**	**↔**	**↔**
					10, 30 mg/kg bw/d	**▼**	**↔**	**▼**	**↔**
	5748	GD 6	PND 21	GD 6 - PND 21	1.7 mg/kg bw/d	**↔**	**↔**	**↔**	**↔**
					10.2 mg/kg bw/d	**▼**	**↔**	**▼**	**↔**
					30.6 mg/kg bw/d	**▼**	**↔**	**▼**	**▲**
	6748	GD 6	PND 22	GD 6 - PND 21	0.1mg/kg bw/d	**↔**	**↔**	**↔**	**↔**
					15, 30 mg/kg bw/d	**▼**	**↔**	**▼**	**↔**
	2218	GD 1	PND 21	GD 1 - PND 21	0.3 mg/kg bw/d	**↔**	**↔**	**↔**	**ND**
					3 mg/kg bw/d	**▼**	**↔**	**↔**	**ND**
					30 mg/kg bw/d	**▼**	**▲**	**▼**	**ND**
Perchlorate	18235	GD 6	PND 21 (pups), PND 30 (dams)	GD 6 - PND 30	30 ppm	**↔**	**↔**	**▼**	**↔**
					300 ppm	**▼**	**↔**	**▼**	**↔**
					1000 ppm	**▼**	**↔**	**▼**	**▲**
Triclosan	1657	GD 6	DG 20	GD 6 - PND 21	10, 30, 100 mg/kg bw/d	**↔**	**↔**	**↔**	**↔**
					300 mg/kg bw/d	**▼**	**↔**	**▼**	**↔**
			PND 21 (pups), PND 22 (dams)		10, 30 mg/kg bw/d	**↔**	**↔**	**↔**	**↔**
					100, 300 mg/kg bw/d	**↔**	**↔**	**▼**	**↔**
Cyanamide	7478	GD 7	GD 15 (dams) PND 16 (pups)	GD 7 - PND 22	7.5, 11.25 mg/kg bw/d	**↔**	**↔**	**↔**	**↔**

Symbols and abbreviations as in **Table 5**.

### 3.7 Relation of response patterns to modes of action of thyroid hormone system perturbances

To investigate whether the observed T4 – TSH response patterns can be related to documented modes of action (MOA) of the tested chemicals, we compiled the relevant information for each of the four response patterns for all studies, including single-dose experiments (**Table 7**). This included histological analyses of the thyroid gland, expression profiles of genes coding for TH axis markers, enzymatic activities, TH-related enzymatic activities and protein levels, T4 levels in the brain and other neurological outcomes (see materials and methods for the name of the assays and **Supplementary Materials 6, 7** for the detailed extracted data). Because most studies did not provide MOA data themselves or reported such data incompletely, we extracted additional relevant information from the CompTox Chemical Dashboard database. We excluded 2 studies with the phthalates DHP and DCHP (758, 2863) due to the unclear responses of T4/TH. Both phthalates altered the thyroid histomorphology.

**Table 7:** T4 – TSH response patterns of chemicals and their modes of action.

↓T4, ↑TSH Chemical (Cad. Ref. Num)	NIS inhibition	TPO inhibition	Distributor proteins; TH transporters	Liver enzyme induction				Xenobiotic-sensing receptors						DIO (and IYD) inhibition		TRs		TSHr		TRHr		THRSP	Thyroid histology	Brain T4	Other
				T4UDPGT (1a1)	UGTs (other)	SULTs	CYPs	CAR			PXR Ag		AhR Ag	Enz. Act.	Gene	Ant	Ag	Ant	Ag	Ant	Ag				
								Ag	Ant																
BPA (2146)	+	+		1a1↔	1a6↔	2a1 ↔	1a1↔	+	↔		+		↔	IYD ↔		+	↔	↔	+	↔	↔	↔			
TCDD (*3121*, 191, 738)				*1a1↔*	+1A6 *+1a6 + 1a7*		+1A1 *+1a1*	+	↔		↔		+			↔	↔	↔	↔	↔	↔		Hyperplasia		+TSH (Pit.)
Amitrole (7478)	+	+ +						↔	↔		↔		↔			↔	↔	↔	↔	↔	↔				
PFBS (2343 in mice)																									*↑Trh*
Chlorpyrifos (2118)	+	+			1a6↔	2a1↔		+	↔		+		+			+	↔			↔	↔	↔	Necrosis, vacuolation		
Fipronil (*29594*) (dams)	+							↔	+		+		↔	+1,2,3		+	↔	↔	↔	↔	↔				Liver ind.
PTU (930, *5493*, *1205*, AD3, AD4, AD5, AD6, AD7)		+ +	*Mct8↔* *+Oatpc1c* *+Slc7a3* (brain)	1a1↔	1a6↔	2a1↔		↔	+		+		↔	+1	*+1* *2*↔ *(Brain)*	+	↔	↔	↔	↔	↔	↔		↓	↓T4 (liver) *Tra ↔* *↓Trb*
DEHP (527, *544*)				1a1↔	1a6↔	2a1↔		↔	↔		↔		↔		*+3*	↔	↔	↔	↔	↔	↔	↔			+PAX8 *+Pax8* +TTF1 *+Ttf1*
Triclosan (304, 563 in mice)	+	+		1a1↔		2a1↔	1a2*↔*	↔	+		+		↔	+1,2,3 +IYD		+	↔	↔	↔	+	+	↔			
Indomethacin (dams) (*27596*)				1a1↔	1a6↔	2a1↔	1a1*↔* 1a2*↔*	↔	↔		↔		↔			↔	↔	↔	↔	↔	↔	↔			
Perchlorate (dams) (18235, A2)			*Pendrin?*					↔	↔		↔		↔		*1*↔	↔	↔	↔	↔	↔	↔			↓	*Nkx2.1↔* ↓*Pax8* Tg*↔* *↓Trb* *↓Tshr*
Dimethoate (*2261*)*		+		1a1↔	1a6↔	2a1↔	+1a1 +1a2	↔	↔		↔		↔	+IYD		↔	↔	↔	↔	↔	↔	↔			
BDE-209 (1172)*								↔	↔		↔							↔	↔	↔	↔				
↓T4, TSH↔ Chemical (Cad. Ref. Num)	NIS inhibition	TPO inhibition	Distributor proteins; TH transporters	Liver enzyme induction				Xenobiotic-sensing receptors						DIO (and IYD) inhibition		TRs		TSHr		TRHr		THRSP	Thyroid histology	Brain T4	Other
				T4UDPGT (1a1)	UGTs (other)	SULTs	CYPs	CAR			PXR Ag		AhR Ag	Enz. Act.	Gene	Ant	Ag	Ant	Ag	Ant	Ag				
								Ag	Ant																
TBBPA (1456)	+	+			1a6↔		1a1↔ 1a2↔	↔	↔		+		↔	+1,2,3 +IYD		+	↔	↔	↔	+	+	↔			
Aroclor 1254 (2275, *3441*, 1947, *1981*)				?										2? (brain)									↓Area follicle	↓	
PCB 77 (1126)				*+*																					
PCB 153 (2215)								↔	↔		↔		↔			+	↔	↔	↔	↔	↔				
4-OH-CB107 (3341, *2367*)			TTR	1A1↔										1↔ (Liver)										↓	PROD↔ EROD↔ (Liver)
PFOS (2295, 1765, 1247)	+	↔					1a1↔	↔	↔		+		↔	+2 +3		+	↔	↔	↔	↔	↔				
PFHxS (2429, *27706*)	*Nis↔*	*Tpo↔*	*Slc3a23 ↔*	*1a1↔*		*+1c3*	*+2b1* *1a1↔* *3a11↔* *3a23↔*								*1*↔							↔	Small or no persistent	↓	*Nkx2.1↔* *Mdra1↔* *Me1↔* *Tshr↔* *Car*↔ *Pxr*↔
ETU (166)		+ +		1a1↔	1a6↔	2a1↔	1a1↔ 1a2↔	↔	↔		↔		↔			↔	↔	↔	+	↔	↔	↔	↓ Area follicle		
Vinclozolin (106, 3257)				1A1↔	1a6↔		CYP p450↔	↔	↔		+		↔			↔	↔	↔	↔	↔	↔	↔			+BROD +EROD +MROD Liver ind.
PBDE-47 (6748)								+	↔		+		↔			+	↔	+	↔	↔	↔				
DE-71 (930, 5748, 6748, 2218, *A1*)															*↓1*					↔	↔		Vacuolation		T4*↔* (liver)
DBDE (14143)								↔	↔		↔		↔					↔	↔	↔	↔				
Nitrofen (*2043*)	+					2a1 ↔		↔	↔		+		↔			↔	↔	↔	↔	↔	↔				
T4 ↔, TSH ↔ Chemical (Cad. Ref. Num)	NIS inhibition	TPO inhibition	Distributor proteins; TH transporters	Liver enzyme induction				Xenobiotic-sensing receptors						DIO (and IYD) inhibition		TRs		TSHr		TRHr		THRSP	Thyroid histology	Brain T4	Other
				T4UDPGT (1a1)	UGTs (other)	SULTs	CYPs	CAR				PXR Ag	AhR Ag	Enz. Act.	Gene	Ant	Ag	Ant	Ag	Ant	Ag				
								Ag		Ant															
HBCD (2320)								↔		+		+	↔	1↔		+	↔	+	↔	↔	↔		Hypertrophy		
Selenium (22710)								↔		↔		↔	↔			↔	↔	↔	↔	↔	↔				
Pentacholorophenol (*1920*)	+	+						+		↔		+	+			+	↔	↔	↔	+	+				*TRb?*
Ethanol (*22532*)								↔		↔		↔	↔			↔	↔	↔	↔	↔	↔				
Glyphosate (267)			*↓Mct8, ↓Oatp1c1* *(Hyp.)* *+Mct8* *+Oatp1c1* (Pit.) *Mct8↔* (Liver)												*+2,3 (Hyp.)* *1,3↔ (Liver)*										*Tshb↔ (Pit.)* *+TRa1* *+TRb1* *(Pit., liver)*
PCB 118 (*1820*)																									EROD*↔*
Potassium iodide (*11193*)* - ↓ft4	*Nis ↔*		*Mct8 ↔* *Duox↔* *Pendrin↔*					↔		↔		↔	↔		*2 ↔*	↔	↔	↔	↔	↔	↔		No effects		*Tg ↔*
PCB 126 (1126)				+1A1																					
Cyanamide (7478)		+		1a1↔	1a6↔	2a1↔		↔		↔		↔	↔			↔	↔	↔	↔	↔	↔	↔			
Imidacloprid (*4842 in mice*)				1a1↔				↔		↔		↔	↔			↔	↔	↔	↔	↔	↔				TR Binding affinity
Mancozeb (*4842 in mice*)		+ +						↔		↔		+	↔	+1,2,3		+	↔	↔	↔	↔	↔				TR Binding affinity
Monocrotophos (*27438*)		+		1a1↔	1a6↔	2a1↔		↔		↔		↔	↔			↔	↔	↔	↔	↔	↔	↔	Irregular follicular shape		
2-MP (7478)		+						↔		+		+	+			↔	↔	↔	↔	↔	↔				
Methimazole (dams) (*27238*)	↔	+		1a1↔	1a6↔	2a1↔		↔		↔		↔	↔			↔	↔	↔	↔	↔	↔		Irregular follicular shape		
Genistein (14584)		+ +		1a1↔	1a6↔	2a1↔		+		↔		+	+	+1 IYD↔		+	↔	↔	↔	↔	↔	↔			

Patterns of hormonal changes are depicted as follows: decreased T4 and increased TSH (↓T4, ↑TSH), decreased T4 and unaltered TSH (↓T4, **↔**TSH), unaltered T4 and different effects for TSH (**↔**T4, ? TSH), unaltered T4 and TSH (**↔**T4, TSH). Data sources for MOA information are: the present systematic review (blue), CompTox Chemicals Dashboard (black), EFSA report for thyrotoxicity of pesticides (16). Names of test compounds are listed with Cadima Reference Numbers (parentheses). Cadima Numbers in green are for Tier 1 studies (Definitely or probably low risk of bias), in black Tier 3 studies (Definitely or probably high risk of bias). Thyroid hormone system endpoints are organized as follows: synthesis of TH (NIS and TPO inhibition), distributor proteins and TH transporters, liver enzyme induction (T4-UDPGT, UGTs, SULTs, CYPs) and xenobiotic-sensing receptors (CAR Agonist (Ag), CAR Antagonist (Ant), PXR Ag, AhR Ag), deiodinases inhibition (DIO and IYD), thyroid receptors (TR Ag and TR Ant), TSH receptors (TSHr Ag and Ant), TRH receptors (TRHr Ag and Ant), Thyroid Hormone Responsive (THRSP), thyroid histology, levels of T4 in brain and other markers: TSH and T4 levels in brain and/or liver, BROD, EROD, MROD, PAX8, TTF1, NKX2.1, TG, MDRA1, ME1, “liver induction”, TR binding activity and gene expression of TR/TSHr. When a chemical induced a positive response, this was marked with a “+”; no effect of the test compound “↔” and decreased response with a “↓”. Chemicals without a clear response are marked with “?”. Readouts extracted from the systematic review (blue) are written in capitals letters for enzymatic activity/protein levels, and in italics for gene expression. Other abbreviations: pituitary (pit.), hypothalamus (hyp.).

Chemicals which produced T4 decrements with concomitant increases in TSH were frequently capable of inhibiting TH synthesis (**Table 7**). Examples are BPA, amitrole, chlorpyrifos, fipronil, PTU, triclosan and dimethoate. Based on data extracted from *in vitro* assays (CompTox), there are indications that BPA, TCDD, chlorpyrifos, fipronil, PTU, triclosan and dimethoate also affect hepatic metabolism by interacting with xenobiotic-sensing receptors (CAR, PXR) and inducing mono-oxygenases (CYP) and conjugating enzymes that glucuronidise TH (UGT). PTU, fipronil, DEHP, triclosan and dimethoate were able to suppress the enzymatic activity or gene expression of DIOs and IYD. PTU and perchlorate also led to decreases in T4 brain concentrations in offspring. Pax8 and Ttf1, transcription factors associated with thyroid development and involved in the regulation of thyroid-related genes, were also altered after DEHP (527) and perchlorate (AD2) exposures.

The chemicals that produced T4 decrements without corresponding TSH increases were frequently able to act as PXR agonist. This is the case for TBBPA, PFOS, Vinclozolin, BDE-47 and Nitrofen. Only PCB 77 was capable of inducing T4 UDPGT (or UGT1A1), which catalyzes T4-glucuronidation. In addition, vinclozolin tended to induce other hepatic enzymes as BROD, EROD and MROD. Interestingly, 4-OH-CB107 showed binding affinity for TTR.

The case of TBBPA is interesting, as its *in vitro* effect profile would be expected to produce T4 – TSH response patterns different from the observed serum T4 decrements without attendant TSH increases. As an inhibitor of TH synthesis, TBBPA might be expected to also produce TSH increases, while its capability to antagonise the TR would lead to T4 increases with no TSH changes.

A similar pattern would be expected from PFOS, due to its ability to antagonise TRs, while its ability to inhibit NIS would be expected to produce T4 decrements and TSH increases, different from the pattern observed (T4 decrements, no TSH increase).

Following the approaches in (30, 31), we next classified our test compounds according to their capacity to interfere with TH regulation (TH receptors, TRH, TRH receptors, TSH, TSH receptors), TH synthesis (NIS, TPO), TH binding proteins (TTR), TH transporters (MCT8, OATP), control of local TH action (DIO/IYD), and induction of hepatic enzymes (UGTs, SULTs, CYPs, AhR, PXR, CAR, PPARa) (See **Table 8** in **Supplementary Material 12**). This classification shows that several chemicals were able to interact with more than one target of the TH system, e.g. BPA, Aroclor 1254, genistein or chlorpyrifos. Scarce data are available for other markers, such as TH transporters or binding proteins.

## 4 Discussion

We present a comprehensive overview of scientific reports characterizing changes in T4 and TSH serum levels observed after gestational exposures of rodents to various chemicals affecting the thyroid hormone system. By employing generic terms in our search strategy, excluding specific chemical names, we captured over 30,000 records which we subjected to relevance screening. Some relevant records may have been missed because search terms were not present in title or abstract, but we believe this number to be small.

Our evidence mapping shows that studies of T4 and TSH changes after gestational exposures are limited to a relatively small set of chemicals in which pesticides, pharmaceuticals and industrial chemicals are somewhat under-represented. A wider range of substances has been examined in studies of adult exposures (to be reported elsewhere).

The T4 and TSH changes we detected were not significantly affected by issues of between-study reproducibility. Similar changes were observed in multiple independent studies of the same chemical, and any divergent findings could be explained in terms of differences in the administered doses, the duration of dosing or the timing of TH measurements. There was reasonably good concordance between dams and pups, and any differences are likely due to differences in toxicokinetics. Additional factors that can come into play include sensitivity differences between pup and dam, due to their developmental stage which may blur concordances in some cases. The lactational transfer of some chemicals may also confound concordances.

Considerable variations in the timing of hormone measurements became apparent, especially in offspring, and greater consideration should be given to harmonization of timing. Many studies limited hormone measurements in pups to the end of lactation (PND 21) which may have missed some effects, as more pronounced alterations in T4 and TSH are often observed earlier (GD20, PND 4 or 14).

Our risk-of-bias analysis exposes deficiencies in TH and TSH analytics as a potential problem. For most eligible studies, we were unable to exclude bias, mainly because of inadequate descriptions of method details, or because of the use of human immunoassays on rat sera without adaptation or validation.

One aim of this exercise was to support future hazard assessments by producing insights of the spectrum of MOAs involved in TH system disruption. However, most of the studies considered here measured only a few endpoints useful for elucidating potential MOAs. Accordingly, we had to make inferences about MOAs from extraneous sources, which may compromise their validity due to complications with *in vitro* to *in vivo* extrapolations and differences in exposure time scales (32–35).

With these provisos in mind, the following patterns became apparent: Gestational exposures to certain chemicals during the time when the thyroid develops can lead to disruptions of the HPT feedback loop, with increased TSH levels in dams and offspring. Our evidence mapping shows that chemicals acting in this way include amitrole, bisphenol A, chlorpyrifos, DEHP, PTU, TCDD and triclosan. A feature common to this group is a capacity to interfere with TH synthesis by inhibiting iodide transport into the thyroid (NIS inhibition) and/or by inhibiting TPO, based on evidence from *in vitro* assays.

Similar disruptions of the HPT feedback mechanism do not occur with other chemicals also capable of suppressing circulating TH levels after gestational exposures, even though the observed TH decrements often exceed those seen with chemicals that disrupt the HPT axis. Substances that fall into this group include PCBs, PBDEs, PFAS, TBBPA and vinclozolin. With a few exceptions, these chemicals are not able to interfere with TH synthesis. Instead, their MOA appears to be best characterized as induction of microsomal enzyme systems in the liver via PXR and/or CAR which promote the elimination of TH, by glucuronidation.

Other patterns observed after gestational exposures include elevated T4 levels but without TSH changes, consistent with TRb antagonism, and increases in TSH with no changes in T4, suggestive of TH transmembrane transport inhibition (e.g. MCT8). Finally, there were T4 increases accompanied by TSH increases, a phenotype observed in DIO2 knock-out mice (36).

As pointed out in the Results section, the phenotypes expected based on *in vitro* test outcomes do not always materialize *in vivo* (an example are the patterns expected from the TR antagonist properties of bisphenol A). In cases where substances interact with multiple molecular targets, the prediction of *in vivo* phenotypes from *in vitro* results becomes particularly complex.

The current EU approaches for identifying THSDC rely on determinations of altered TH serum levels as indicators of a hormonal mode of action and on demonstrations of histopathological changes in the thyroid gland required as indicators of adversity. Since thyroid histopathological changes are driven by TSH, chemicals that produce TH alterations without accompanying TSH increases will be missed with the current implementation of the EU criteria. Our evidence mapping shows that this applies to many of the substances for which data after gestational exposures are available. This is of concern as it may lead to overlooking chemicals that produce developmental neurotoxicity by disrupting adequate TH supply to the brain, but without increasing TSH. Examples are Aroclor 1254 (4) and DE-71 (37) which produced changes in motor activity in rat offspring. In both cases, strong T4 decreases in offspring were observed, but TSH levels were left unchanged. Similarly, Aroclor 1254 impaired hearing in offspring (4), again without any TSH changes. These studies suggest that it is the extent of T4 decrements, and not TSH, that is an important predictor of developmental neurotoxicity. Whether this applies to all indicators of developmental neurotoxicity is currently unclear. Periventricular heterotopia, the occurrence of misplaced neurons due to migration deficits, were found in studies with PTU or amitrole which produces T4 decrements (38, 39), but were not seen after gestational PFHxS or triclosan exposures, despite pronounced reductions in T4 serum levels (8).

However, these considerations do not apply to the regulatory regimes in countries outside the EU where endocrine disruptors are not treated differently from other chemicals and where endocrine disruptor criteria do not exist.

In any case, we show that T4 decrements in offspring can arise from a multitude of MOAs. In view of recent evidence of a direct role of T4 in the brain via non-genomic signaling (40), these observations are particularly interesting.

However, TSH increases do not seem to be linked to a similar multitude of MOAs. Disruptions of the HPT axis after gestational exposures appear to be linked to inhibition of TH synthesis and can occur even after relatively modest declines of TH serum levels. In a review of mechanisms of drug metabolizing enzyme inducers and TH alterations, Vansell (41) has drawn attention to another MOA leading to TSH increases. In studies of liver microsomal enzyme system inducers, the most pronounced disruptions of the HPT axis occurred with substances that promoted T3 glucuronidation and transmembrane transporters important in the biliary excretion of TH conjugates, such as Mrp2. Sometimes, strong TSH increases occurred even after only modest T4 serum decreases such as with clobazam which induced UGT1A1/*Ugt1a1* (T4 glucuronidation), UGT2B/*Ugt2b1, Ugt2b2* (T3 glucuronidation) and MRP2/*Mrp2* (biliary excretion of TH conjugates) (42, 43). Our evidence mapping shows that the relevant TH conjugating enzymes and transmembrane transporters are rarely measured in studies of hormonal changes, but more information on these enzyme systems may be helpful in further elucidating the MOAs behind T4 – TSH response patterns.

## Data availability statement

The original contributions presented in the study are included in the article/**Supplementary Material**, further inquiries can be directed to the corresponding author.

## Author contributions

IF-P: Conceptualization, Data curation, Formal analysis, Investigation, Methodology, Visualization, Writing – original draft, Writing – review & editing. AHB: Conceptualization, Data curation, Formal analysis, Investigation, Methodology, Visualization, Writing – original draft, Writing – review & editing. AK: Conceptualization, Formal analysis, Funding acquisition, Supervision, Writing – original draft, Writing – review & editing.
